# The NICOLA recruitment trial (NICOLA-RT): can you improve recruitment by making zero cost amendments to the invitation letter?

**DOI:** 10.1186/1745-6215-16-S2-P106

**Published:** 2015-11-16

**Authors:** Lisa Maguire, Frances Burns, Mike Clarke

**Affiliations:** 1 Queen's University Belfast, Belfast, UK; 2 University of Liverpool, Liverpool, UK

## Background

NICOLA-RT is using minor edits to invitation letters to assess their impact on potential participants’ decisions about joining a prospective study. Even small improvements in recruitment could produce important savings in cost and time. The elements in NICOLA-RT have been registered as SWAT (Study Within A Trial) 3, 4 and 5. These SWAT examine different approaches to recruitment and retention and ways to maximise these.

## Aims and objectives

NICOLA-RT examines the impact of differing invitation letters on study recruitment, with the following specific objectives: assessing if the gender of the person signing the letter affects recruitment; assessing the impact of describing the research as a ‘study’ or ‘project’ and assessing if including an explicit statement about confidentiality for the participants in the invitation letter has an impact.

## Methods

The study is a 3x2x2 factorial randomised trial of different invitation letters. It is embedded in the ongoing NICOLA cohort study, and tests three different signatures (male, female and the trial group), the use of ‘study’ versus ‘project’ and the presence or absence of an explicit statement about confidentiality.

## Results

Recruitment for the NICOLA cohort began in January 2014 and is expected to continue until late 2015. Although the cohort is still recruiting, this presentation will detail preliminary findings from the interim analysis of more than 7700 letters, which have revealed no significant differences to date.

## Conclusion

Recruitment is continuing until NICOLA recruits 8,500 participants and a final analysis of NICOLA-RT will be conducted at that stage.

## Trial Registration

NCT01938898

